# Magic state injection on IBM quantum processors above the distillation threshold

**DOI:** 10.1038/s41598-026-40381-1

**Published:** 2026-02-26

**Authors:** Younghun Kim, Martin Sevior, Muhammad Usman

**Affiliations:** 1 School of Physics, The University of Melbourne, Parkville, 3010 VIC, Australia; 2 Data61, CSIRO, Clayton, 3168 VIC, Australia

**Keywords:** Engineering, Mathematics and computing, Physics

## Abstract

The surface code family is a promising approach to implementing fault-tolerant quantum computations. Universal fault-tolerance requires error-corrected non-Clifford operations, in addition to Clifford gates, and for the former, it is imperative to experimentally demonstrate additional resources known as magic states. Another challenge is to efficiently embed surface codes into quantum hardware with connectivity constraints. This work simultaneously addresses both challenges by employing a qubit-efficient rotated heavy-hexagonal surface code for IBM quantum processors (ibm_fez) and implementing the magic state injection protocol. Our work reports error thresholds for both logical bit- and phase-flip errors, of $\approx 0.37\%$ and $\approx 0.31\%$, respectively, which are higher than the threshold values previously reported with traditional embedding. The post-selection-based preparation of logical magic states $|H_L\rangle$ and $|T_L\rangle$ achieve fidelities of $0.8806\pm 0.0002$ and $0.8665\pm 0.0003$, respectively, which are both above the magic state distillation threshold. The post-selection process yields an average success rate of $36.28 \pm 0.09\%$. Additionally, we report the minimum fidelity among injected arbitrary single logical qubit states as $0.8356\pm 0.0003$. Our work demonstrates the potential for realising non-Clifford logical gates by producing high-fidelity logical magic states on IBM quantum devices.

## Introduction

Quantum error correction is vital to achieving scalable and universal fault-tolerant quantum computation by suppressing inevitable errors in contemporary quantum computing^1–4^. Surface codes have emerged as one of the leading quantum error correction protocols, which promise to protect quantum information by encoding it across many entangled qubits^5–8^. They stabilize qubits and focus errors into relatively easy-to-correct forms and require a straightforward coupling map^9^. In recent years, surface code implementations, albeit on a small scale, have been demonstrated on a variety of quantum hardware platforms such as superconducting^10–13^, trapped ion systems^14^, and neutral atom systems^15^. However, research on surface code implementations on quantum processors remains at a preliminary stage and universal fault-tolerant quantum computing is still an open problem that requires demonstrations of a full surface code protected universal gate set including both Clifford and non-Clifford quantum operations^16–18^.

Although the experimental realization of Clifford gates with surface code formalism and its variants has been quite successful in the literature^15,19–25^, it is well known theoretically that the implementation of non-Clifford operations is a highly nontrivial task that requires employing a specific logical qubit state, known as a logical magic state^19,26^. Therefore, the experimental realization of the logical magic state is an important milestone towards achieving universal quantum computing^27–30^, which is a topic of ongoing research. Only two experimental studies^29,30^ have reported preparing magic states using surface codes. While Ref. ^29^ has shown distance-3 implementations exceeding the distillation threshold value for square lattice code, the study in Ref.^30^ was only distance-2 surface codes without scaling. In this work, we report the first distance-3 implementations of surface code-based magic state injection on an IBM quantum processor and demonstrate that any arbitrary single logical qubit state can be prepared, including logical magic states above the distillation threshold. Additionally, our work designs a rotated surface code embedding in the heavy-hexagonal architecture of IBM quantum processors. This is also the first such implementation on IBM devices and reduces the physical qubit requirements by approximately half compared to previous surface code embeddings^31,32^, thus significantly reducing the resource requirements for scalable surface code implementations.

Our code exhibits a relatively high threshold compared to other lattice-compatible codes^32–35^. We obtain $\approx 0.37\%$ and $\approx 0.31\%$ threshold values for both logical bit- and phase-flip errors, respectively. Additionally, we conduct magic state injection experiments using distance-3 codes on the ibm_fez device. Our circuit can encode any arbitrary single-qubit state into the logical qubit state of the code. Among logically encoded states, the logical magic states relevant for the non-Clifford quantum operations namely $|H_L\rangle$ and $|T_L\rangle$ are prepared with fidelities of $0.8806\pm 0.0002$ and $0.8665\pm 0.0003$, respectively, both exceeding their distillation threshold values. To achieve this goal, we use post-selection, which has an average success rate of $36.28 \pm 0.09\%$.

**Fig. 1:**
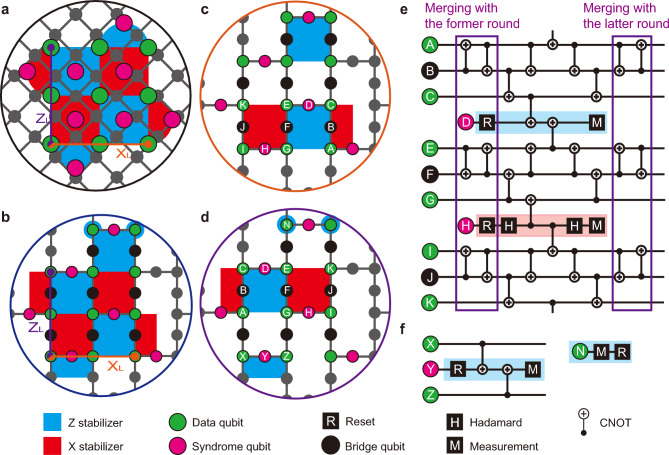
Embedding rotated surface code. (**a**) and (**b**) Qubit arrangements of the rotated surface code in the square and heavy-hexagon lattices. The logical Z and X operators are represented by the purple and orange solid lines, respectively. (**c**) and (**d**) The stabilizers of the code are divided into two sub-groups. (**e**) A sub-round syndrome extraction circuit measures weight-four stabilizers including those at the side boundaries within the same sub-group. The first and last two layers of CNOT gates can be executed in parallel along with the former and latter sub-rounds. (**f**) Subroutine circuits for measuring weights-two and -one Z stabilizers at top and bottom boundaries.

## Results

### Rotated surface code embedding

The rotated surface code is an optimized variant of the conventional surface code that reduces resources while preserving code distance, by using a different orientation of qubits. We note that, throughout this work, we use the term distance or code distance to refer to its standard definition, the minimum number of non-trivial Pauli operators that preserve the code subspace. Previous work has implemented surface codes in a heavy-hexagon lattice^32^, but did not embed its rotated version. This work investigates the feasibility and efficiency of embedding the rotated surface code while respecting connectivity limitations. We estimate the threshold value for logical Pauli errors as a function of code distance and demonstrate encoding logical magic states in the rotated version of surface codes on one of IBM’s devices.

**Fig. 2:**
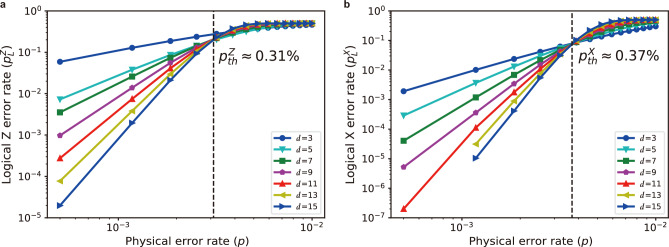
Logical error rates. (**a**) and (**b**) Logical Z and X error rates as functions of the code distance, denoted as *d*, and the physical error rate (*p*), showing error suppression via scaling code size. While the code distance ranges from 3 to 15, the physical error rate *p* varies from $5\times 10^{-4}$ to $10^{-2}$. The logical error rates are calculated using $5\times 10^6$ samples. The threshold values are obtained as $p^Z_{\text {th}}\approx 0.31\%$ and $p^X_{\text {th}}\approx 0.37\%$ for logical Z and X errors, respectively.

In a square lattice, the rotated surface code displayed in Fig. 1a can concurrently stabilize data qubits in a subspace that yields +1 eigenvalues for its stabilizer group. The stabilizer group includes weight-four at the bulk and weight-two stabilizers at the boundaries, defined with commutable multi-qubit Pauli operators. However, the limited connectivity of the heavy-hexagon lattice makes embedding of rotated code a challenging task. This is because each qubit has at most three neighbors instead of four, which limits measuring weight-four stabilizers in the same manner as on the square lattice. The rotated surface code can be embedded in the heavy-hexagon lattice, as shown in Fig. 1b, by stabilizing data qubits through two consecutive sub-rounds. The qubit layout used to embed larger-distance codes is shown in Supplementary Fig. 2. Each sub-round measures the corresponding subgroup stabilizers, as illustrated in Fig. 1c and d.

1a $$\hat{Z}_{A} \hat{Z}_{C} \hat{Z}_{E} \hat{Z}_{G} \underset{{{\mathrm{unfold}}}}{\overset{{{\mathrm{fold}}}}{\longleftrightarrow}}\hat{Z}_{C} \hat{Z}_{E},$$

1b $$\hat{Z}_{E} \hat{Z}_{G} \hat{Z}_{I} \hat{Z}_{K} \underset{{{\mathrm{unfold}}}}{\overset{{{\mathrm{fold}}}}{\longleftrightarrow}}\hat{Z}_{G} \hat{Z}_{I},$$

The key idea behind measuring weight-four stabilizers, such as $\hat{Z}_A\hat{Z}_C\hat{Z}_E\hat{Z}_G$ and $\hat{X}_E\hat{X}_G\hat{X}_I\hat{X}_K$, while respecting the connectivity limitation is transforming them into weight-two stabilizers, in other words, folding stabilizers. The folded stabilizers are then measured using syndrome qubits to extract their eigenvalues. Following measurement, the original stabilizers are restored by reversing the series of quantum gates applied during the folding process, which is unfolding stabilizers. Figure 1e shows a sub-round syndrome extraction circuit for the measurement of weight-four stabilizers following the process of (un)folding stabilizers as expressed in (1a) and (1b) as detailed in Ref.^32^. Further demonstration is provided in Supplementary Fig. 3, which visualizes the (un)folding stabilizer process. The circuit requires long-range interactions between data qubits, which can be done effectively by employing bridge qubits as intermediaries. To optimize the circuit, the first and last two layers of CNOT gates in the former and latter sub-rounds are applied simultaneously to minimize the circuit depth across sub-rounds.

The main difference between the rotated and unrotated surface code on the heavy-hexagon lattice is that the rotated version adopts weight-2 stabilizers at the boundaries. These stabilizers are formulated to be measured in a way that commutes with the syndrome extraction protocol used for weight-4 stabilizers. While the weight-2 stabilizers at the side boundaries can also be measured with those at the bulk, the weight-2 stabilizers at the top or bottom boundaries need to be measured as weight-two and -one stabilizers as illustrated in Fig. 1f. For the Z stabilizers at the bottom boundary, the two-weight stabilizers can be directly measured using the syndrome qubits. For those at the top boundary, we update the stabilizer group by measuring weight-four or weight-one stabilizers, such as $\hat{Z}_N$, as sub-rounds executed alternatively. The measurement of the weight-one stabilizers collapses the corresponding data qubit states, allowing the remaining data qubits to span the code space where qubit information is protected from errors. Since the measurement of these stabilizers does not involve bridge qubits, they require relatively few time steps compared to those required for weight-four. However, we simultaneously apply measurements for stabilizers in the same sub-round regardless of their weight.

**Table 1:** The required number of physical qubits for embedding rotated and unrotated surface codes on the heavy-hexagon lattice as a function of the code distance (*d*).

	Unrotated	Rotated
$\#$ of data qubits	$2d^2-1$	$d^2+d-1$
$\#$ of qubits	$5d^2 -2(d+1)$	${5/2}d^2 + 2d - {7/2}$

To evaluate the code, we computed logical error rates as a function of the physical error rates under the circuit-level uniform noise model (the details are in Methods). Figure 2a and b show the logical error rates for the two states, either in the X ($|+_L\rangle$) basis or in Z ($|0_L\rangle$) basis, corresponding to the probability of logical Z and X errors, respectively. The logical error rates are calculated under various physical error rates (*p*) ranging from $5\times 10^{-4}$ to $10^{-2}$ and code distances (*d*) ranging from 3 to 15. Although the number of qubits required for the rotated code scales with the code distance as $O(d^2)$, the same as for its unrotated version, the former requires about half the number of qubits in the limit of large code distances. The number of qubits as a function of the code distance is listed in Table 1.

**Fig. 3:**
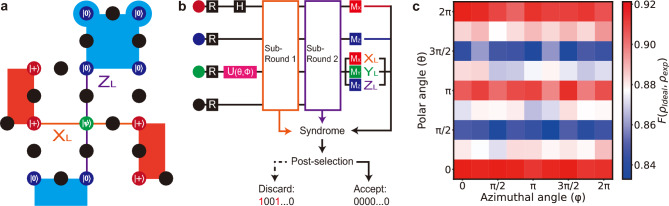
Magic state injection and implementation. (**a**) The initialization layout for magic state injection utilizing the embedded rotated surface code. Each data qubit is initialized with the ground state in either the Z basis ($\left| {0}\right\rangle$), represented as blue nodes, or the X basis ($\left| {+}\right\rangle$), represented as red nodes. The central data qubit, magic state, is prepared in the state $|\psi \rangle$ and depicted as a green node. (**b**) The data qubits are initialized according to the layout, employing a single-qubit unitary operation (U) for the magic state. Following that, two consecutive sub-round syndrome extraction circuits are executed. The magic state is measured in the basis determined by the logical Pauli measurement, while the remaining data qubits are measured in the same basis as their initialization. The outcomes are used to detect errors and discard non-trivial syndromes through post-selection. (**c**) The fidelities of raw logical magic states prepared using the circuit (**b**) are plotted on a plane with the polar ($\theta$) and azimuthal ($\phi$) angles, ranging from 0 to $2\pi$. The values are estimated from the trivial syndromes obtained by sampling $2 \times 10^4$ times for logical measurements in each Pauli basis.

**Fig. 4:**
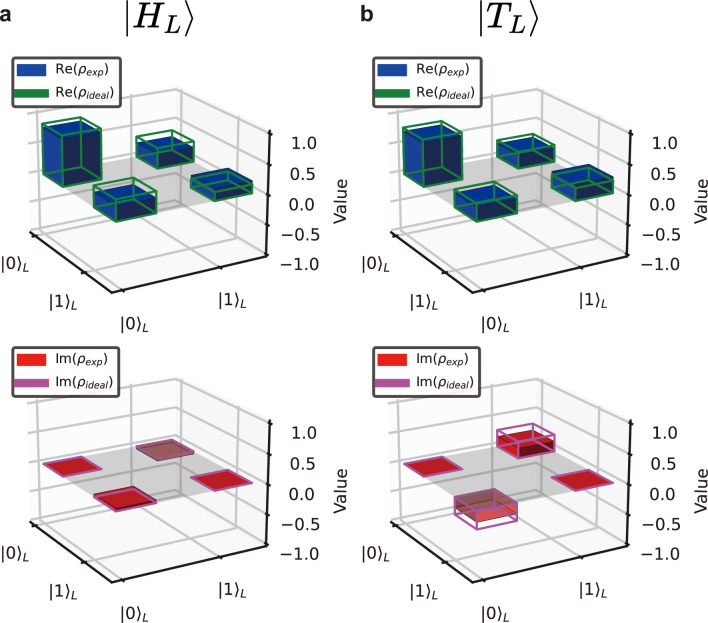
Density matrices of logical magic states. (**a**) and (**b**) The real and imaginary values of the density matrices for the ideal and experimental results are plotted for $|H_L\rangle$ and $|T_L\rangle$, with ideal values represented by lines and experiment values by bars. A grey plane indicates the region where the value is 0.

We estimate the threshold value under uniform circuit-level noise by decomposing the noise into probabilistic Pauli gates characterized by a single physical error parameter (*p*). The resulting threshold values for logical phase- and bit-flip errors are approximately $0.31\%$ and $0.37\%$, respectively. Although its unrotated version has a threshold of $0.3\%$^32^, the highest among other lattice-compatible codes suggested so far, we find that the proposed code in this work achieves a slightly even higher threshold value for both error types. Notably, we observe a faster improvement in performance for bit-flip error corrections compared to phase-flip error corrections as the physical error rate decreases below the threshold, given the same code distance. Additionally, the threshold value for a logical Z error is smaller than that for a logical X error. This indicates an asymmetric feature, that has not been observed in the square lattice, in correcting the two types of errors under the unbiased noise model when rotating the embedded code in the heavy-hexagon structure.

One particular class of errors in syndrome measurement circuits is known as hook errors, in which a single fault can escalate into multiple data-qubit errors. Such errors can arise due to two-qubit interactions, as is the case in our code when protecting against phase-flip errors. The number of correctable errors is at most $\lfloor \frac{d+1}{2}\rfloor$, given the code distance *d*^36^. In practice, especially when prioritizing scalability and real-time feedback, not all such errors can be corrected if suboptimal syndrome extraction circuits or decoders are used^7,32^. As a result, the presence of hook errors or limitations of algorithmic decoders can degrade its practical performance. To reflect the impact of such circuit-level faults, we use the term the effective code distance ($d_{eff}$) following Refs.^37^ and^38^. Our numerical results, illustrated in Fig. 1a and b, show that while the rotated surface code on a heavy-hexagon array preserves the code distance for bit-flip errors, with $d_{eff}^X = d$, the distance for phase-flip errors is degraded, following $d_{eff}^Z \sim d/2$. More details on the effective distance have been discussed in the Supplementary Section I.

### Magic state injection protocol

We next discuss a code-based magic state injection protocol. The protocol consists of four main steps: 1. Initialization, 2. Stabilizer measurement, 3. Logical Pauli measurement, and 4. Post-selection. Previous works have demonstrated a magic state injection protocol for a square array, which relies on two rounds of syndrome extraction to detect errors^39,40^. Following Ref.^29^, we present a variant adapted for embedding on a heavy-hexagon array, using only a single round of the syndrome extraction circuit to minimize the circuit depth and reduce the adverse effects of measurement errors, as follows: (1) Initialization: Data qubits are prepared in their designated quantum states, as depicted in Fig. 3a. These states include the ground state of the Z basis ($|0\rangle$) or the X basis ($|+\rangle$). Additionally, the central qubit, the magic state, is prepared as $|\psi \rangle =\text {cos}(\theta /2)|0\rangle + e^{i\phi }\text {sin}(\theta /2)|1\rangle$, intended for injection at the logical qubit level, using a primitive single-qubit gate parameterized with $\theta$ and $\phi$. (2) Stabilizer measurement: Two sub-rounds of syndrome extraction circuits are performed and measure full stabilizers. (3) Logical Pauli measurement: As shown in Fig. 3b, the central data qubit is measured in the same Pauli basis as the interrogated logical Pauli measurement basis i.e. X, Y, or Z. In contrast, the remaining data qubits are measured in the same basis as initialized. (4) Post-selection: Based on the measured outcomes, we evaluated deterministic parity values to produce a syndrome. When there is an error, a non-trivial syndrome is created and therefore discarded, as shown in Fig. 3b. Specifically, the measurement outcomes from the sub-round syndrome extraction circuits should align with the initially conditioned stabilizers at the boundaries, and the parity of the data qubit measurements associated with the boundary stabilizers needs to match the eigenvalue of the corresponding stabilizer. Numerical results of the magic state injection protocol are provided in Supplementary Fig. 4.

We decided to design the protocol using a distance-3 rotated surface code, which reduces the number of qubits by one-third compared to its unrotated version. The reduced qubit requirement minimizes errors and increases the success rate for the post-selection experiments. The data qubits are initialized to have eigenvalues of +1 at all boundary stabilizers. They are then stabilized through the sub-round syndrome extraction circuits. In the absence of noise, these processes map the data qubits into the codespace, forming a logical qubit. Depending on the state injected into the center data qubit using predefined parameters $\theta$ and $\phi$ in the initialization process, the state of the logical qubit is prepared as $|\psi _L\rangle =\text {cos}(\theta /2)|0_L\rangle + e^{i\phi }\text {sin}(\theta /2)|1_L\rangle$. When we measure the logical qubit on a logical Pauli basis, we estimate the measurement by the parity of the measured outcomes from the data qubits associated with the target basis, where $\hat{Y}_L=i\hat{X}_L\hat{Z}_L$^29^. We can change the basis of this projective logical Pauli measurement by measuring the magic state on a target basis.

We conducted experiments using 25 physical qubits on ibm_fez quantum device, one of IBM’s quantum processors, accessed via the cloud. The experiments were conducted using the parameters $\theta$ and $\phi$ ranging from 0 to $2\pi$ in $\pi /4$ intervals. The details of the preparation of an arbitrary magic state ($|\psi \rangle$) through a primitive single-qubit gate are provided in Methods. Each logical Pauli measurement for the X, Y, and Z basis is repeated $2\times 10^4$ times per state with samples taking approximately $6.8\mu$s. The syndrome extraction circuits analyzed in this work consist of 78 two-qubit gates and 19 qubit measurements. All circuits employ conditional reset gates immediately following measurement to reinitialize qubits.

It is worth discussing the error rates of logic quantum gates, particularly two-qubit gates and hardware measurements. During our experiments, the average error rate from the two-qubit gates ($2.9\times 10^{-3}$) was below the threshold values for both types of code logical errors. In contrast, the same for the readout case ($1.6\times 10^{-2}$) was an order of magnitude higher compared to the threshold values. Further details of the error rates can be found in the Methods. Although this might make logical qubits more susceptible to measurement-induced logical errors than gate-induced ones, the post-selection in the experiments could effectively detect both measurement-induced and gate-induced errors during error detection, potentially improving the fidelity of logical magic states. As a result, the average acceptance rate for experiments for each logical Pauli measurement that met the post-selection criteria was $36.28\pm 0.09\%$. In Methods, we show the acceptance rates of post-selection in eigenstates of logical Pauli operators.

2 $$\begin{aligned} F(\rho _{\text {ideal}},\rho _{\text {exp}}) = \left( Tr\left( \sqrt{\sqrt{\rho _{\text {exp}}}\rho _{\text {ideal}}\sqrt{\rho _{\text {exp}}}}\right) \right) ^2 \end{aligned}$$

Based on the post-selected samples from the experiments on the IBM device, we calculate fidelities for logical qubit states. The fidelities are calculated between the ideal ($\rho _{\text {ideal}}$), and experimentally reconstructed density matrix ($\rho _{\text {exp}}$) as expressed in Eq. (2). While the ideal density matrix is obtained from the theoretical logical state of the qubit as $\rho _{\text {ideal}}=|\psi _L\rangle \langle \psi _L|$, the experimental density matrix is estimated based on the expectation values of the logical Pauli operators (see Methods). Furthermore, we compare theoretical and experimental logical Pauli expectation values in Supplementary Fig. 1.

In Fig. 3c, we plot the fidelities for the experimentally encoded logical qubit states. When the polar angle ($\theta$) is an integer multiple of $\pi$ regardless of the azimuthal angle ($\phi$), logical qubits are prepared in the eigenstates of the logical Z operator where the values are prominently high. However, we observe a gradual decrease in the fidelities of qubit states, when the state is injected as the superposition of two computational Z basis states with different phases. Under the assumption that primitive gates in the hardware have no biased noise, we attribute the relative vulnerability of phase information to the inherent bias of the resilience of the code against bit-flip errors. This aligns with the asymmetric feature of the code analyzed theoretically. Furthermore, the minimum value has been found in the Y basis state with +1 eigenvalue from the logical Y operator as $0.8356\pm 0.0003$, and the state is susceptible to both bit-flip and phase-flip errors. However, even with antisymmetric robustness against errors, we note that our protocol achieves an average fidelity of $0.882\pm 0.006$.

Finally, we test our model in the preparation of well-known logical magic states, $|H\rangle = \cos (\pi /8)|0\rangle + \sin (\pi /8)|1\rangle$ and $|T\rangle = \cos (\beta )|0\rangle + e^{i \pi /4}\sin (\beta )|1\rangle$, where $\cos (2\beta )=1/\sqrt{3}$. H- and T-type states can be used to realize phase-shift gates, which belong to non-Clifford gates^26^. The threshold fidelity values of $|H\rangle$, using a 7-to-1 distillation routine^16^, and $|T\rangle$, using a 5-to-1 distillation routine^26^, are 0.854 and 0.827.

We conduct experiments to prepare the magic states and analyze their fidelities. The results are shown in Fig. 4a and b where we compare the ideal and experimental density matrices for the two logical magic states. The fidelity of the logical magic states, $|H_L\rangle$ and $|T_L\rangle$, are prepared with the fidelity $0.8806\pm 0.0002$ and $0.8665\pm 0.0003$, respectively, which are above the threshold for the distillation protocol. The uncertainty of fidelities is estimated using a bootstrapping technique (see Methods).

## Discussion

In this work, we prepare an arbitrary encoded logical single-qubit state using a rotated surface code on the ibm_fez quantum processor. First, we demonstrate a rotated surface code, which requires around half the number of qubits as its unrotated version for large code distances in the heavy-hexagon structure of ibm_fez. We compute the threshold values of the code and find an asymmetric feature in error correction. We have achieved high fidelity using the injection protocol on arbitrary single-qubit states for logical encoding. We also realize two logical magic states $|H_L\rangle$ and $|T_L\rangle$ type, which can be employed to implement non-Clifford gates for quantum error correction. During the demonstration, we employ post-selection to achieve high-fidelity magic states, with an average yield of $36.28 \pm 0.09\%$. The results show we exceed the threshold fidelity of magic state distillation on IBM hardware.

Several challenges remain to achieve the universality of the logical quantum gate set; however, our work marks an important step towards universal quantum computing by demonstrating the preparation of raw logical magic states on an IBM quantum device. In future work, it would be intriguing to conduct quantum memory experiments and demonstrate error suppression using our scheme, using code distances from 3 to 5, and even 7, by shaping the code as rectangular on 156 physical qubit devices. Note that achieving error suppression requires sufficiently low measurement error rates, as the syndrome is only partially extracted over time. Another challenge is mitigating hook errors, which degrade the effective code distance. Employing flag qubits can be a plausible solution, as the information they provide can help construct distance-preserving codes, where the code distance and effective code distance are equal^32,38^. Furthermore, increasing the size of the logical magic states and implementing lattice surgery would be another promising avenue for future work^40,41^. In summary, our protocols pave the way for promising near-term advancements in quantum error codes for quantum hardware with connectivity constraints such as the heavy-hexagon structure employed by the IBM quantum processors.

## Methods

### Noise model

We evaluate threshold values of the rotated surface code embedded in the heavy-hexagonal structure under a circuit-level noise model. We adopt the noise model that decomposes error channels using Pauli operators. A depolarizing error channel is used, where the errors are not biased but have a uniform probabilistic distribution among the Pauli errors^42^. When the error rate is *p*, the circuit-level noise model consists of the following noisy channels: (1) Single-Qubit Depolarizing Error Channel: A single qubit subjected to the error channel experiences Pauli errors ($\hat{X}$, $\hat{Y}$, and $\hat{Z}$) with equal probabilities. The error probabilities for $\hat{X}$, $\hat{Y}$, and $\hat{Z}$ are denoted by $p_X$, $p_Y$, and $p_Z$, respectively, satisfying the condition $p_X = p_Y = p_Z = p/3$. This occurs when a physical qubit is inactive and undergoing free evolution or when a single-qubit gate, such as $\hat{H}$, is applied. (2) Initialization and Measurement Error Channel: A bit-flip error, with a probability of *p*, is applied before measurement (*M*) and after the reset gate (*R*) on the basis Z. (3) Two-Qubit Depolarizing Error Channel: Two qubits are susceptible to Pauli errors when a two-qubit gate (CNOT) is applied. These Pauli errors of two qubits are represented by the set $\{\hat{X}, \hat{Y}, \hat{Z}, \hat{I}\}^{\otimes 2}/\{\hat{I} \otimes \hat{I}\}$. The probability of each error is uniform as *p*/15.

### Calculating threshold

In this work, to compute a logical error rate, we turn measured results from syndrome extraction circuits into a syndrome to detect errors and use it to calculate a correction operator. Errors are detected through flipped measurement outcomes from the same syndrome qubits, which will produce “1” bits in the syndrome, indicating the presence of errors between the circuit rounds for weight-four and -two stabilizers. However, results from weight-one stabilizers are directly used to detect errors. We use the open-source software tool Stim to generate syndrome samples using sub-round syndrome extraction circuits under circuit-level noise, decomposing noise as probabilistic Pauli gates^43^. Furthermore, we use Pymatching to determine a correction operator as the most likely error based on the noise model^44,45^. A logical error rate is computed as the ratio of the average number of rounds to have a logical error for varying error rates and code distances.

**Fig. 5:**
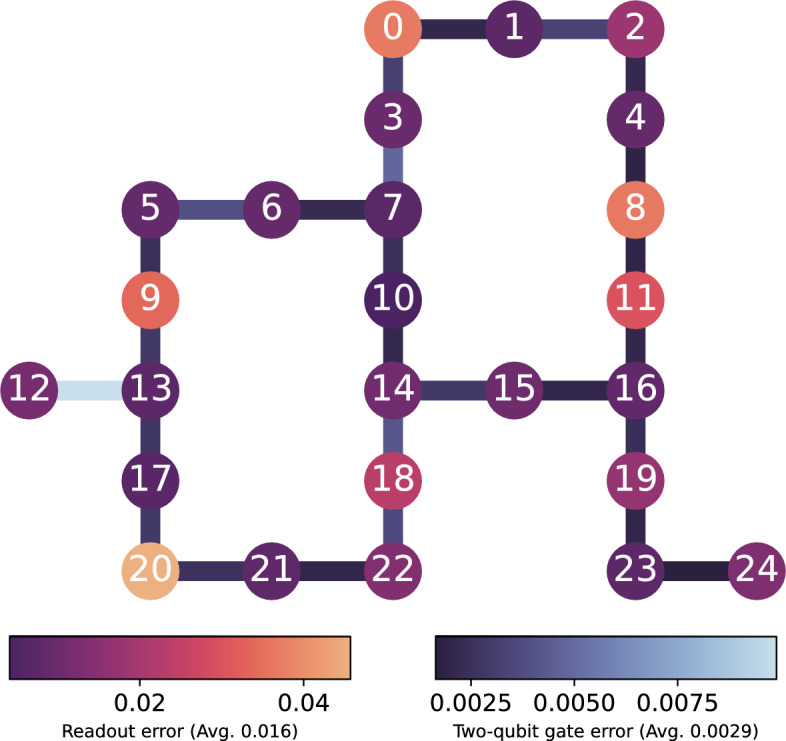
Hardware specifications. The graph shows the chosen physical qubits’ specifications of the distance-3 rotated surface code onto the heavy-hexagon structure ($\texttt {ibm\_fez}$). Each node and edge correspond to the physical qubit and the connectivity of a two-qubit gate (CZ). The error rates of readout and two-qubit gate are displayed with colors. Their average error rate are $1.6 \times 10^{-2}$ and $2.9 \times 10^{-3}$.

### IBM hardware

We conducted the experiments on 31st October 2024, utilizing 25 out of 156 physical qubits on ibm_fez device. Measurement and two-qubit gate (CZ gate) error rates may vary per qubit within the device’s configuration. The error rates for the chosen qubits in the experiments are the hardware’s calibration data and are illustrated in Fig. 5. The average error rates for readout and two-qubit gates were $1.6 \times 10^{-2}$ and $2.9 \times 10^{-3}$, respectively.

### Implementation of the experiment

We conducted optimized quantum circuits for the magic state injection protocol on the hardware. First, to optimize circuits, data qubits forming weight-one stabilizers are measured only once rather than twice, because there are redundant measurements of data qubits in the second sub-round syndrome extraction circuit and logical Pauli measurement. Second, we classically controlled the X gates based on the measurement results of the qubits to reset the physical qubits^46^. This approach reduced the cost of syndrome extraction circuits by minimizing the time to reset qubits. We applied dynamic decoupling to the physical qubits while inactive, to minimize unwanted perturbations during quantum operations, including measurements^47^.

We used a U3 gate to rotate a single qubit to prepare the $|\psi \rangle$ state from the ground state ($|0\rangle$), where the gate takes three parameters $\theta$, $\phi$, and $\lambda$. The gate represents:

$$\begin{aligned} U3(\theta ,\phi ,\lambda ) = \begin{pmatrix} \text {cos}(\theta /2) & -e^{i\lambda }\text {sin}(\theta /2)\\ e^{i\phi }\text {sin}(\theta /2) & e^{i(\phi +\lambda )}\text {cos}(\theta /2)\end{pmatrix}, \end{aligned}$$

where we have set $\lambda = 0$. We employed the Python library Qiskit to transpile circuits into the device’s basis gates, enabling the execution of quantum circuits on the hardware^48^.

### Qubit tomography

Any single-qubit density matrix ($\rho$) can be written in terms of its Pauli operators $\vec {\sigma }=(\hat{X}, \hat{Y}, \hat{Z})$ and the identity operator ($1\!\!1$), such that:

3 $$\begin{aligned} \rho = \frac{1}{2}(1\!\!1+\vec {\sigma }\cdot \vec {r}) = \begin{pmatrix} 1-z & x-iy\\ x+iy & 1+z \end{pmatrix}, \end{aligned}$$

where $\vec {r} = (x, y, z)$ is a real vector representing the Bloch coordinates of $\rho$. The Bloch vector corresponds to the coefficients of each Pauli operator and can be used to reconstruct the density matrix. A single-qubit tomography is a process to estimate the Bloch vector based on the outcomes of non-commuting observables. The simplest method, direct inversion tomography, repeatedly measures a qubit state in the Pauli bases and obtains the expectation value for each basis, reconstructing the density matrix of the target single-qubit state^49^.

The numbers of repeated measurements along the logical Pauli X, Y, or Z basis can be denoted by $N_X$, $N_Y$, and $N_Z$, respectively. Each measurement yields one of two outcomes: “up-state” with a +1 eigenvalue for the corresponding Pauli operator, or the “down-state” corresponding to a $-1$ eigenvalue. The counts of the up- and down-states are represented as $N_{up}$ and $N_{down}$ and their sum is the total number of measurements for a particular Pauli basis. For example, $N_Z = N_{|0\rangle }+N_{|1\rangle }$, and similarly for the other Pauli bases, $N_X$ and $N_Y$. Based on these measured outcomes, the Bloch vector ($\vec {r}_{\text {exp}}$) can be estimated by the expectation value of each Pauli operator as follows^49^:

4 $$\begin{aligned} \vec {r}_{\text {exp}} = \left( \frac{N_{|+\rangle }-N_{|-\rangle }}{{N_{X}}}, \frac{N_{|+i\rangle }-N_{|-i\rangle }}{{N_{Y}}}, \frac{N_{|0\rangle }-N_{|1\rangle }}{{N_{Z}}} \right). \end{aligned}$$

**Table 2:** Acceptance rates of post-selection in different Pauli bases, where $N=2\times 10^4$.

Logical state	$N_X/N$	$N_Y/N$	$N_Z/N$
$|0\rangle _L$ ($\theta =0,\phi =0$)	0.3643	0.3704	0.3619
$|1\rangle _L$ ($\theta =\pi ,\phi =0$)	0.3643	0.3685	0.3548
$|+\rangle _L$ ($\theta =\pi /2,\phi =0$)	0.3581	0.3615	0.3511
$|-\rangle _L$ ($\theta =\pi /2,\phi =\pi$)	0.3713	0.3674	0.3677
$|+i\rangle _L$ ($\theta =\pi /2,\phi =\pi /2$)	0.3616	0.3715	0.3633
$|-i\rangle _L$ ($\theta =\pi /2,\phi =3\pi /2$)	0.3669	0.3734	0.3613

$N_X$ is the number of samples passed the post-selection for measuring logical X measurement. Likewise for $N_Y$ and $N_Z$.

As mentioned in the main paper, logical Pauli measurements are repeated $2\times 10^4$ per injected magic state. We discard any sample which has a non-trivial syndrome, it may vary the number of samples for $N_{up}$ and $N_{down}$ states. The acceptance rates of experimental results for the eigenstates of Pauli operators are listed in Table 2. The samples that passed the post-selection are then used to calculate expectation values of logical Pauli operators corresponding to logical magic states.

### Bootstrapping

The confidence intervals in experimental data are estimated using a bootstrapping method^50^. We classically resampled using the probability distribution obtained from the experiments.

## Supplementary Information

Supplementary Information.

## Data Availability

All datasets are available in the manuscript figures. Further data and source code can be available in the Code Ocean capsule at https://codeocean.com/capsule/4035970/tree
