# Supplementary material for: Magic state injection on IBM quantum processors above the distillation threshold
Source: Sci Rep. 2026 Feb 26;16:11189. doi: 10.1038/s41598-026-40381-1 (PMC13046941; doi:10.1038/s41598-026-40381-1)
Supplement: Supplementary file 1 — Supplementary Information. [file 41598_2026_40381_MOESM1_ESM.pdf]

# Supplementary information for "Magic state injection on IBM quantum processors above the distillation threshold"

## I. ASYMMETRIC FEATURE OF THRESHOLD

Fig. 2a and b in the main text show logical error rates of Z and X errors, respectively. There is a crossover point for each logical error type, known as the threshold value ($p_{\text{th}}$), where the relation between the physical error rate and the corresponding logical error rate changes: For $p < p_{\text{th}}$, $p_L$ decreases as the code distance increases, whereas for $p > p_{\text{th}}$, $p_L$ increases with the code distance. The threshold values for logical phase- ($p^Z_{\text{th}} \approx 0.31\%$) and bit-flip ($p^X_{\text{th}} \approx 0.37\%$) errors are distinct.

$$p^Z_L = C_Z/\Lambda_Z^{a_Z(d+1)/2}, \quad p^X_L = C_X/\Lambda_X^{a_X(d+1)/2} \tag{S1}$$

To analyze in more detail, we use the fitting equations (S1) describing the logical error rate of the Z and X errors as $p^Z_L(p,d)$ and $p^X_L(p,d)$ [1]. The fitting is carried out using predefined threshold values for phase- and bit-flip errors denoted as $p^Z_{\text{th}}$ and $p^X_{\text{th}}$, respectively, where $\Lambda_Z \propto p^Z_{\text{th}}/p$ and $\Lambda_Z \propto p^X_{\text{th}}/p$. In these formulas, the terms $C_Z$, $C_X$, $a_Z$, and $a_X$ are fitting constants. Especially, $a_Z$ and $a_X$ are parameters closely relevant to the code performance, specifically associated with the number of errors that form a non-trivial error chain. Even in the regime where the qubit error rate is lower than a threshold, which requires exponential growth in the number of samples, these statistical arguments allow estimating logical error rates using fitting values [1–5].

When the parameters ($a_Z$ and $a_X$) are set to 1, the minimum number of errors required to produce a logical error is $\frac{d+1}{2}$ for an odd code distance $d$. This shows how the code can efficiently correct bit-flip and phase-flip errors. However, if these parameters are less than 1, it indicates that a logical error could occur with fewer errors. For example, with a code distance of $d = 3$, at least two errors are required to cause a logical error. However, if one of the parameters is less than 1, even a single error could induce a logical error of that type, indicating the possibility of a weight-one error transforming into a weight-two error.

$$a_Z \approx 0.7, \quad a_X \approx 1$$

We obtain the fitting parameters as $a_Z \approx 0.7$ and $a_X \approx 1$ based on the numerical values shown in Fig. 1a and b in the main paper. Although the code to correct for logical X errors maintains the effectiveness of code distance, it is biased and vulnerable to logical Z errors. We note the asymmetric property in correcting for the two types of errors, even under the unbiased noise model. We attributed this property to the propagation of errors among data qubits, so-called a hook error.

Challenges arise in syndrome measurements that involve correlations across multiple qubits for the practical purpose of rectifying errors. A particular type of error, known as a hook error, degrades the performance of quantum error correction because it can cause more than a weight-one error in the syndrome measurement circuit. While the number of correctable errors is at most $\lfloor \frac{d+1}{2} \rfloor$, given the code distance ($d$), there is a case where not all the same-weight errors can be successfully corrected due to hook errors. Therefore, this effect, caused by hook errors, limits the correctable errors and becomes a dominant factor in determining the inferior error-correction performance. Such errors can be observed in our syndrome extraction circuit against phase-flip errors at the circuit-level noise. We use another term the "effective code distance" to refer to the circuit-level distance ($d_{eff}$).

When layers of CNOT gates are implemented in a sub-round syndrome extraction circuit, errors on data qubits can spread through the gates. Crucially, this error propagation occurs, propagating an error from one data qubit to its vertically adjacent neighbor rather than a horizontally neighboring data qubit. The propagation of errors in the horizontal direction is restricted because of the existence of either syndrome qubits or the constraints of direct interaction. In contrast, errors can propagate vertically due to the process for (un)folding stabilizers requiring long-range CNOT gates among vertically neighboring data qubits. This directional limitation on error propagation affects the efficiency of code-correcting errors, as a weight-one error can escalate into a weight-two error. It is important to note that the detrimental impact is particularly significant for a logical error that coincides with the direction of the data qubits associated with the logical operator, weakening the code's error correction capability in such cases. In our case, the propagation of vertical error hampers the correction of a vertically defined logical error, which is the logical Z operator. Therefore, error propagation among data qubits causes ambiguity in calculating a correction operator and a lower error threshold, decreasing the effective code distance ($d^Z_{eff}$) against phase-flip errors.

Due to the dynamical time feature of the syndrome extraction and interaction directions between data qubits, the effective code distance for the logical operator $Z_L$ cannot be preserved in the heavy-hexagon lattice when embedding a rotated surface code, leading to uneven reduction. Based on our numerical data and the fitting parameters ($a_Z$, $a_X$), we expect that the effective code distance for bit-flip errors remains unchanged, with $d^X_{eff} = d$, while for phase-flip errors, the effective code distance is approximately halved in the regime $p \to 0$ and under code distance scaling (i.e. $d^Z_{eff} \sim d/2$).

## II. EXPECTATION VALUES

Fig. S1a shows experimentally estimated logical expectation values of logical Pauli operators. The values are estimated from the samples that produce the trivial syndrome obtained by sampling $2\times10^4$ times for logical measurements on each Pauli basis. Their ideal values can be expressed in terms of polar($\theta$) and azimuthal($\phi$) angles as shown in Fig. S1b:

$$\langle\psi_L|\hat{X}_L|\psi_L\rangle = \sin\theta\cos\phi \quad \text{(S2)}$$

$$\langle\psi_L|\hat{Y}_L|\psi_L\rangle = \sin\theta\sin\phi \quad \text{(S3)}$$

$$\langle\psi_L|\hat{Z}_L|\psi_L\rangle = \cos\theta \quad \text{(S4)}$$

Although the experimental results range approximately from $-0.6$ to 0.6 for the logical X and Y operators and around $-0.8$ to 0.8 for the logical Z operator, their theoretical values vary from $-1$ to 1. However, we find that the experimental results closely align with the ideal values.

## III. ROTATED SURFACE CODE ON A HEAVY-HEXAGON ARRAY

In this section, we briefly explain how the code can scale and demonstrate how the syndrome extraction circuit (un)folds stabilizers. Fig. S2 illustrates how the stabilizers and qubits are assigned to embed distance-3, 5, and 7 codes. We formulate hardware requirements and find that a distance-$d$ code requires $d^2+d-1$ data qubits, $(d^2+2d-3)/2$ syndrome qubits, and $d^2-1$ bridge qubits. In total, embedding the distance-$d$ code needs $5/2d^2+2d-7/2$ physical qubits as discussed in the main text.

With respect to the syndrome extraction circuit, we describe how stabilizers can be transformed from weight-4 ($\hat{Z}_A\hat{Z}_C\hat{Z}_E\hat{Z}_G$, $\hat{X}_E\hat{X}_G\hat{X}_I\hat{X}_K$) to weight-2 ($\hat{Z}_C\hat{Z}_E$, $\hat{X}_G\hat{X}_I$) through a folding process, and vice versa through unfolding. We begin with Pauli gate propagation rules through a CNOT gate.

$$\hat{Z}_A\hat{Z}_C\hat{Z}_E\hat{Z}_G \underset{\text{unfold}}{\overset{\text{fold}}{\rightleftarrows}} \hat{Z}_C\hat{Z}_E \quad \text{(S5a)}$$

$$\hat{X}_E\hat{X}_G\hat{X}_I\hat{X}_K \underset{\text{unfold}}{\overset{\text{fold}}{\rightleftarrows}} \hat{X}_G\hat{X}_I \quad \text{(S5b)}$$

The X operator propagates from the control qubit to the target qubit, while the Z operator propagates in the reverse direction, from the target to the control. Fig. S3a and b illustrate how the weight-4 Z and X stabilizers spread across data qubits through layers of CNOT gates involving bridge qubits in the syndrome extraction circuits. To provide a clearer illustration, we use blue and red to indicate the propagation paths of the Z and X operators, respectively. We start by applying $\hat{Z}_A\hat{Z}_C\hat{Z}_E\hat{Z}_G$ in Fig. S3a, $\hat{X}_E\hat{X}_G\hat{X}_I\hat{X}_K$ Fig. S3b. It is important to note that Pauli operators cancel out when applied twice. As a result, after four layers of CNOT gates, the operations on the first and fourth data qubits are eliminated due to gate propagation from the nearest-neighbor bridge qubits. The initial operators are thus transformed into $\hat{Z}_C\hat{Z}_E$ and $\hat{X}_G\hat{X}_I$, respectively, acting on the second and third data qubits. The eigenvalues of the reduced-weight stabilizers are measured by interacting with neighboring syndrome qubits. The operators are then expanded back into weight-4 stabilizers by reversing the previously applied CNOT gates. The (un)folding stabilizer process is repeated twice to measure all syndrome operators defined by the code.

## IV. QUANTITATIVE COMPARISON

Here, we compare our results with those from Ref. [6], focusing on hardware requirements, yields, and fidelities of magic states. Both approaches use a distance-3 surface code, but on different qubit coupling maps. While our code is designed to embed the surface code on a heavy-hexagon structure, the work in Ref. [6] implements the code directly on a square array. Although the heavy-hexagon lattice does not provide optimal connectivity for the surface code, our results are compatible with those obtained using the square lattice.

TABLE S1: Comparison of two magic state injection results using a distance-3 surface code.

| **Reference** | **Ref.[6]** | **Ours** |
|---|---|---|
| Coupling map | Square | Heavy-hexagon |
| # of Data Qubits | 9 | 9 |
| # of Syndrome Qubits | 8 | 6 |
| # of Bridge Qubits | 0 | 8 |
| Fidelity of $\|H\rangle_L$ | $0.9090\pm0.0009$ | $0.8806\pm0.0002$ |
| Fidelity of $\|T\rangle_L$ | $0.8890\pm0.001$ | $0.8665\pm0.0003$ |
| Yield (%) | $73.413\pm0.008$ | $36.28\pm0.09$ |
| Round duration ($\mu$s) | $\approx 4.15$ | $\approx 6.8$ |

To overcome connectivity constraints, our code incorporates additional hardware resources in the form of bridge qubits. As a result, approximately 50% more physical qubits are required to embed the distance-3 surface code (23 physical qubits) on a heavy-hexagon lattice compared to the conventional surface code (17 physical qubits). In addition to the increased qubit count, our scheme also uses an extra round of the syndrome extraction circuit to time-dynamically measure all stabilizers. This leads to a longer overall cycle duration, as measurement operations are the dominant factor in the duration of quantum circuits. Surprisingly, we note that, although the post-selection success rate in our experiments is roughly half that reported in Ref. [6], the fidelities of both H- and T-type magic states remain comparable and exceed their respective theoretical threshold values. We mainly attribute the low yields to the use of

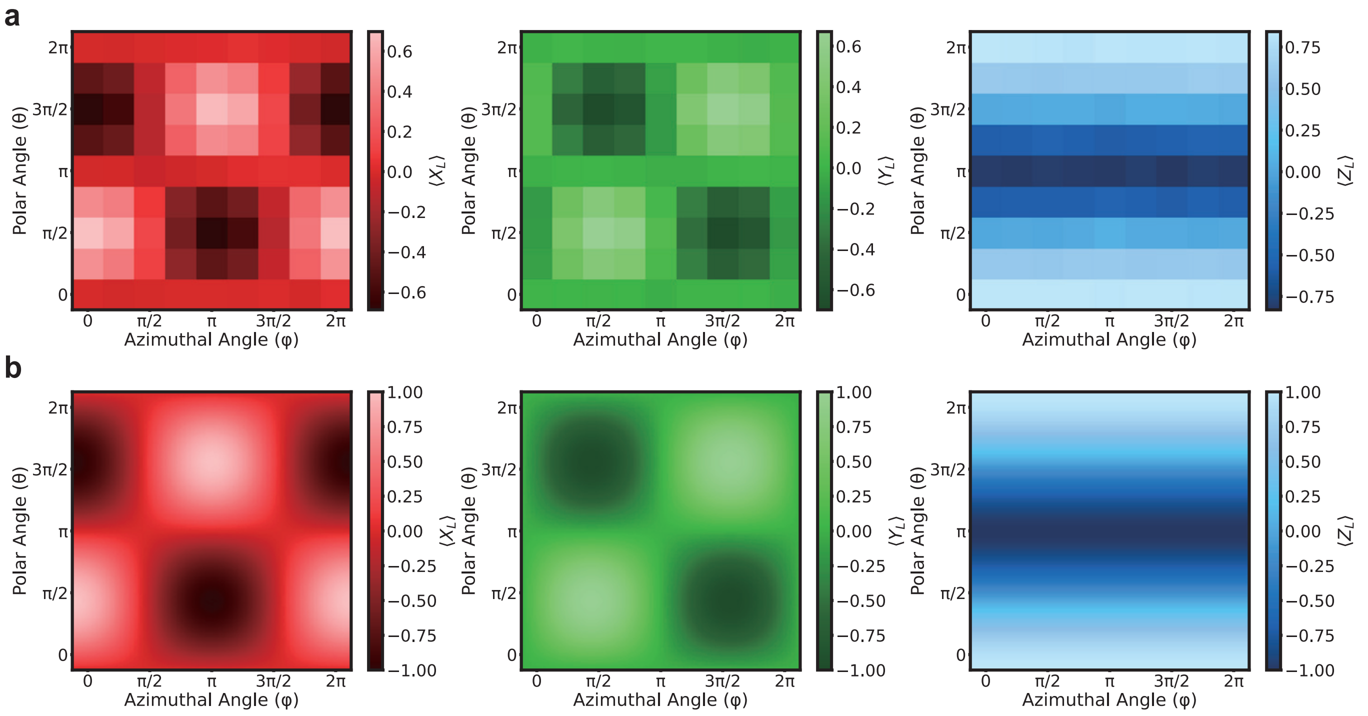


FIG. S1: **Expectation values. a.** The expectation values of logical Pauli operators X, Y, and Z in raw logical magic states prepared on `ibm_fez` device. These values are plotted on a plane with the polar $(\theta)$ and azimuthal$(\phi)$ angles, ranging from 0 to $2\pi$. **b.** Their theoretical values are plotted as functions of parameters $\theta$ and $\phi$ as expressed in (S2), (S3), and (S4).

more primitive gates and the introduction of additional physical qubits for (un)folding stabilizers.

## V. NUMERICAL RESULTS FOR DISTANCE-3 AND DISTANCE-5 CODES

Fig. S4 illustrates the logical error rates for the magic state preparation scheme using distance-3 and 5 codes on a heavy-hexagon array. To compute logical error rates, we use the circuit-level noise model described in the Methods, along with post-selection that accepts only trivial syndromes. For each point, $10^6$ samples have been obtained before post-selection, and confidence intervals at 99.5% are plotted. We estimate logical Z and X error rates by injecting the $|0\rangle$ and $|+\rangle$ states, respectively.

We first compare the logical error rates of both distance-3 and -5 codes with a single syndrome extraction round. The logical error rates against bit-flip errors are higher than those against phase-flip errors, which is consistent with experimental observations. Notably, the impact of increasing the code distance depends on the error type. For example, while scaling the code can reduce logical error rates for bit-flip errors, it makes the code more susceptible to phase-flip errors.

We also compute logical error rates with two rounds of syndrome extraction. Although two rounds are used to detect measurement errors, the impact of increasing syndrome rounds is negligible in both distances as shown in Fig. S4. It implies that the dominant factors causing logical errors without being detected are mainly from single- and two-qubit errors rather than measurement errors, when the error rate of measurement is equal to that of single- and two-qubit gates. Furthermore, the rejection rate for a higher distance code and more syndrome rounds is larger than for a lower distance code and fewer syndrome rounds. Therefore, in scenarios where primitive gate error rates are not sufficiently low, limiting the protocol to a single round of syndrome extraction yields better overall performance.

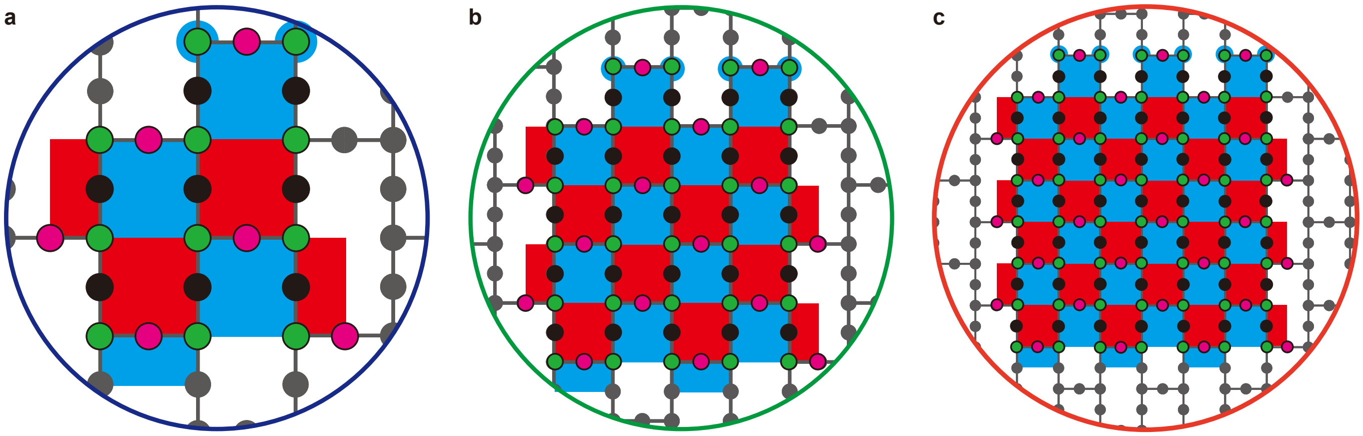


FIG. S2: **Scaling the rotated surface code.** Visualizing qubit arrangements and stabilizers of the rotated surface code in the heavy-hexagon array for **a.** distance-3, **b.** distance-5, and **c.** distance-7 codes.

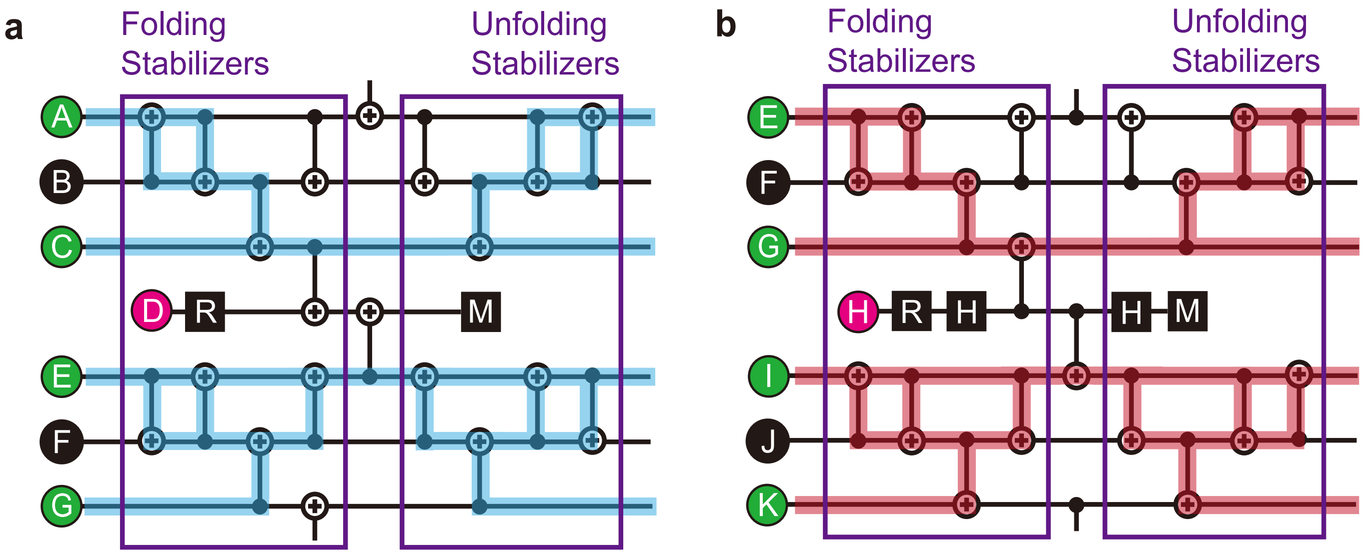


FIG. S3: **(Un)folding stabilizers.** Visualizing (un)folding stabilizer process for the **a.** $\hat{Z}_A\hat{Z}_C\hat{Z}_E\hat{Z}_G$ and **b.** $\hat{X}_E\hat{X}_G\hat{X}_I\hat{X}_K$ stabilizers.

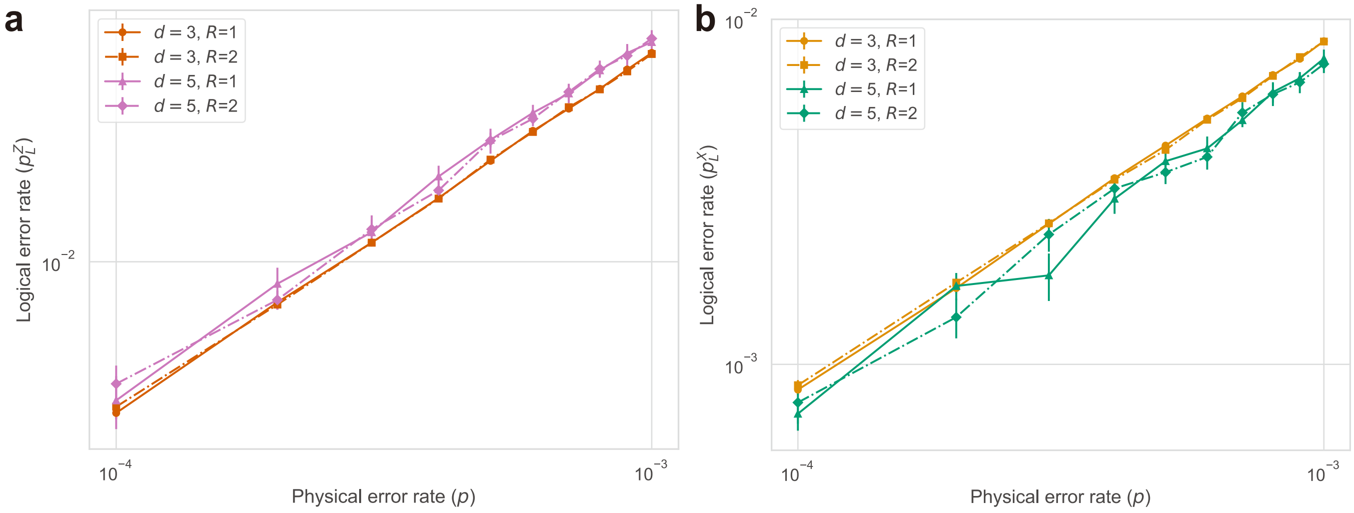


FIG. S4: **Numerical results of magic state injection protocol.** The logical error rates, obtained via post-selection, for the magic state preparation scheme using distance-3 and distance-5 codes on a heavy-hexagon array.

[1] Google Quantum AI *et al.* Exponential suppression of bit or phase errors with cyclic error correction. *Nature* **595**, 383–387 (2021). URL https://www.nature.com/articles/s41586-021-03588-y.

[2] Fowler, A. G., Mariantoni, M., Martinis, J. M. & Cleland, A. N. Surface codes: Towards practical large-scale quantum computation. *Physical Review A* **86**, 032324 (2012). URL https://link.aps.org/doi/10.1103/PhysRevA.86.032324.

[3] Gidney, C., Newman, M., Fowler, A. & Broughton, M. A Fault-Tolerant Honeycomb Memory. *Quantum* **5**, 605 (2021). URL http://arxiv.org/abs/2108.10457. ArXiv:2108.10457 [quant-ph].

[4] Benito, C., López, E., Peropadre, B. & Bermudez, A. Comparative study of quantum error correction strategies for the heavy-hexagonal lattice (2024). URL http://arxiv.org/abs/2402.02185. ArXiv:2402.02185 [quant-ph].

[5] Acharya, R. *et al.* Quantum error correction below the surface code threshold (2024). URL http://arxiv.org/abs/2408.13687. ArXiv:2408.13687 [quant-ph].

[6] Ye, Y. *et al.* Logical Magic State Preparation with Fidelity beyond the Distillation Threshold on a Superconducting Quantum Processor. *Physical Review Letters* **131**, 210603 (2023). URL https://link.aps.org/doi/10.1103/PhysRevLett.131.210603.
